# Neutrophil-lymphocyte ratio complements volumetric staging as prognostic factor in patients treated with definitive radiotherapy for oropharyngeal cancer

**DOI:** 10.1186/s12885-017-3590-0

**Published:** 2017-09-11

**Authors:** Cédric Panje, Oliver Riesterer, Christoph Glanzmann, Gabriela Studer

**Affiliations:** 1 Department of Radiation Oncology, University Hospital Zurich, Rämistrasse 100, CH-8091 Zürich, Switzerland; 2 Cantonal Hospital Lucerne, Spitalstrasse, CH-6000 Lucerne, Switzerland

**Keywords:** Radiotherapy, IMRT, Head and neck cancer, Oropharyngeal cancer, Volumetric staging, Neutrophil-lymphocyte ratio, NLR

## Abstract

**Background:**

Volumetric tumor staging has been shown as superior prognostic tool compared to the conventional TNM system in patients undergoing definitive intensity-modulated radiotherapy (IMRT) for head and neck cancer. Recently, clinical immunoscores such as the neutrophil-lymphocyte ratio (NLR) have been investigated as prognostic markers in several tumor entities. The aim of this study was to assess the combined prognostic value of NLR and tumor volume in patients treated with IMRT for oropharyngeal cancer (OC).

**Methods:**

Data on all consecutive patients treated for locally advanced or inoperable OC with IMRT from 2002–2011 was prospectively collected. Tumor volume was assessed based on the total gross tumor volume (tGTV) calculated by the treatment planning system volume algorithm. The NLR was collected by a retrospective analysis of differential blood count before initiation of therapy.

**Results:**

Overall, 187 eligible patients were treated with a median IMRT dose of 69.6 Gy. Three-year recurrence-free survival (RFS) for low, intermediate, high and very high tumor volume groups was 88%, 74%, 62% and 25%, respectively (p = 0.007). Patients with elevated NLR (>4.68) showed a significantly decreased 3-year RFS of 44% vs. 81% (p < 0.001) and 3-year OS of 56% vs. 84% (p < 0.001). The NLR remained a significant prognostic factor for RFS and OS when tested among tumor volume groups. Univariate and multivariate regression analysis confirmed both tumor volume and NLR as independent prognostic factors. The NLR offered further statistically significant prognostic differentiation of the small/intermediate/large tumor volume groups.

**Conclusion:**

The NLR remains an independent prognostic factor for patients with OC undergoing radiotherapy independent of the tumor volume.

## Background

Definitive intensity-modulated radiotherapy (IMRT) with or without concomitant chemotherapy has been established as standard treatment for locally advanced and inoperable oropharyngeal cancer [1]. Several investigators have previously shown that for non-surgical definitive IMRT collectives of head neck cancer patients volumetric staging may provide a prognostic benefit over the conventional Union for International Cancer Control (UICC) staging system (7th edition) and its T and N categories with regard to all disease control outcome parameters [2–6]. It is known for decades that tumor volume and, in consequence, the number of clonogenic cells is one of the most important predictors for tumor control in radiotherapy [7, 8]. As anatomically (i.e. surgically) defined system, the T and N categories of the standard TNM system are predominantly based on the extent of invasion into adjacent structures, number and site of involved nodes. Included size parameters are one-dimensional diameter measurement, which may not correlate well with tumor volume [9]. Consequently, it has been shown that there is a significant variability in tumor volume and, in consequence, in outcome within a single T category in head and neck cancer [10, 11].

More recently, immunological scores such as the neutrophil-lymphocyte ratio (NLR) have been introduced as prognostic markers for several tumor entities including various sites of head neck cancer [12–17].

Increased blood neutrophils and tumor associated neutrophils have been linked to inferior outcome in cancer [18], particularly the immunosuppressive subset of myeloid-derived suppressor cells [19, 20]. In contrary, several studies have shown that tumor-infiltrating lymphocytes may represent increased anti-tumor immunity with improved local control and long-term prognosis [21–23]. Blood lymphocytes have consequently been identified as significant prognostic marker in head and neck cancer alone as well as part of clinical immunoscores such as the neutrophil-lymphocyte ratio [13].

However, it is not clear yet whether an elevated NLR represents a surrogate parameter for increased tumor burden in advanced disease [24] or rather tumor-associated immunological processes which are mainly volume-independent.

The aim of our study was therefore to explore the correlation between the NLR and the tumor volume in patients with oropharyngeal cancer undergoing definitive IMRT. The hypothesis was that the NLR may offer additional prognostic information to the previously tested volumetric staging system.

## Methods

Data on all consecutive patients with locally advanced or inoperable oropharyngeal cancer (OC) treated with IMRT at our institution from 2002 to 2011 was prospectively collected. Approval of the Local Ethics Committee (Cantonal Ethics Committee Zurich, Nr. 709) is available.

Patients were treated with normofractionated or slightly hypofractionated (2.11 Gy per fraction) definitive IMRT over 6–7 weeks and, if there was no medical contraindication, with weekly cycles of concomitant cisplatin chemotherapy (40 mg/m2/week) or immunotherapy with cetuximab as previously prescribed [3, 25]. Recurrence-free survival and overall survival rates were evaluated. The following clinical parameters were assessed: age at diagnosis, gender, performance status (Eastern Cooperative Oncology Group, ECOG), histology, TN tumor and nodal stage, UICC stage, smoking history, and total tumor volume. Tumor volume was based on the total (nodal and primary) gross tumor volume (tGTV) using information from clinical examination, endoscopy, planning CT as well as magnetic resonance imaging (MRI) and, if available, positron emission tomography (PET) [3]. Tumor volume definition was reviewed by two board-certified authors (GS and CG). Volumetric three-dimensional tGTV measurements in cubic centimeters (cm^3^) were automatically calculated by the treatment planning system volume algorithm (Eclipse® V8.5, Varian Medical Systems, Palo Alto, CA).

Retrospectively collected NLR was obtained from the most recent available differential blood count after diagnosis and before initiation of radiochemotherapy, or, if applicable, before induction chemotherapy by dividing the number of neutrophils by the number of lymphocytes. Neutrophils and lymphocytes were counted in 10^9^/ml. Patients with acute infections, traumatic injuries, or invasive biopsies within two weeks before the blood count were excluded from further analysis.

### Statistics

Statistical analysis was performed using R software (version 3.2) [26] and the packages “survival” and “prodlim”. For comparisons between different groups, the Chi-square and Mann Whitney U test were used. Spearman correlation test was used to analyze correlation between individual factors. Survival analysis was performed using the Kaplan-Meier method and the log-rank test to assess statistical significance. Univariate and multivariate analysis for prognostic factors were investigated using the Cox proportional hazard regression model and the significance level was set to 0.05.

## Results

### Patient and treatment characteristics

Overall, 194 patients treated with IMRT for oropharyngeal cancer at our institution between 2002 and 2011 were identified. Seven patients were excluded due to primary metastatic disease, missing pretreatment differential blood count or inflammatory or traumatic disease within 2 weeks before the pre-IMRT blood count in order to avoid interference with the NLR. Table 1 shows demographic and tumor related characteristics for the remaining 187 eligible patients.

**Table 1:** Patient and treatment characteristics

Parameter	
Age	median 61.6 years (range 36.9–91.4)
Gender	72% male (*n* = 134) 28% female (*n* = 53)
Histology	100% squamous cell carcinoma
Oropharyngeal subsite	52% tonsil (*n* = 97) 40% base of tongue (*n* = 75) 5% vallecula (*n* = 9) 2% soft palate (*n* = 4) 1% posterior wall (*n* = 2)
T stage (UICC 7th edition)	12% T1 (*n* = 22) 31% T2 (*n* = 59) 19% T3 (*n* = 36) 33% T4 (*n* = 61) 5% not available/recurrent disease (*n* = 9)
N stage (UICC 7th edition)	16% N0 (*n* = 30) 12% N1 (*n* = 22) 4% N2a (*n* = 7) 32% N2b (*n* = 60) 29% N2c (*n* = 55) 4% N3 (*n* = 7) 3% not available/recurrent disease (*n* = 6)
UICC Stage (7th edition)	8% Stage II (*n* = 15) 19% Stage III (*n* = 35) 67% Stage IVA (*n* = 122) 4% Stage IVB (*n* = 7) 2% Recurrent disease (*n* = 3)
ECOG performance score	80% ECOG 0 (*n* = 149) 15% ECOG 1 (*n* = 28) 5% ECOG 2 (*n* = 9)
Tumor volume (combined nodal and primary volume); *n* = events (any recurrence)	median 40 cm^3^ (range 3–216 cm^3^); overall 52 events subgroup 1–15 cm^3^: 14% (*n* = 26); 3 events subgroup 15–70 cm^3^: 60% (*n* = 112); 28 events subgroup 70–130 cm^3^: 23% (*n* = 43); 17 events subgroup >130 cm^3^: 3% (*n* = 6); 4 events
Smoking status	active = 62% (*n* = 116) stopped =25% (*n* = 46) never smoked = 13% (*n* = 25)
NLR	median 3.33 (range 0.91–33.71)
IMRT dose prescription	median 69.6 Gy (66–72 Gy) single dose 2–2.11 Gy
Concomitant systemic therapy	42% cisplatin weekly (*n* = 78) 47% reduced number of cisplatin cycles (*n* = 87) 7% cetuximab (*n* = 14) 4% no systemic therapy (*n* = 7)
Induction chemotherapy	8% of patients (*n* = 15)
Follow-up	median 61.2 months (range 1.7–169)

Median IMRT prescription dose to macroscopic tumor was 69.6 Gy (range 66–72 Gy in 30–35 fractions).

### Tumor volume and NLR

Median tGTV was 40 cm^3^ (range 3–216 cm^3^). Based on a previously reported prognostic tumor volumetric staging [2], 14% of the patients (*n* = 26) belong to the low-volume group (<15 cm^3^), 60% (*n* = 112) to the intermediate-volume group (15–70 cm^3^) and 26% (*n* = 49) to the high-volume group (>70 cm^3^). A previously reported forth prognostic subgroup of tumors >130 cm^3^ volume [3] was not separately analyzed due to limited sample size (*n* = 6).

**Fig. 1:**
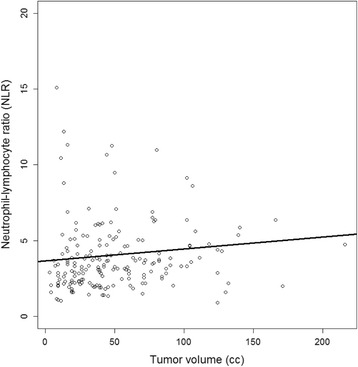
Correlation analysis demonstrates a statistically significant correlation between neutrophil-lymphocyte ratio and total tumor volume (*p* = 0.0059, rho = 0.20)

Median NLR was 3.33 (range 0.91–33.71, lower and upper quartile 2.34 and 4.68, respectively). In the high-tumor volume group (> 70 cm^3^), median NLR was significantly higher than in the low-volume groups with 3.7 versus 3.12 (*p* = 0.035) and NRL correlated significantly with the tumor volume in the whole study population (*p* = 0.006, rho = 0.2, Fig. 1).

### Outcome related to tumor volume and NLR: Recurrence-free survival and overall survival

Recurrence-free survival (RFS) for the entire cohort was 72% at three years and remained unchained at five years. Overall survival (OS) was 77% at three years and 70% at five years, respectively.

**Fig. 2:**
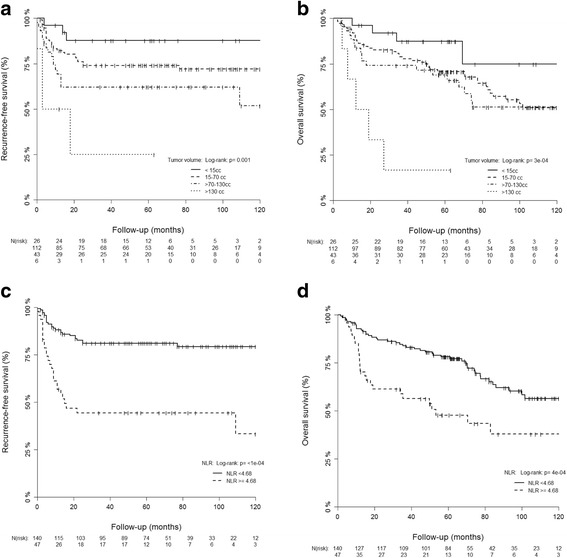
Recurrence-free survival and overall survival is significantly affected by tumor volume group (**a**-**b**) and elevated NLR (> = 4.68) **c**-**d**

Three-year RFS rates were 88%, 74%, 62% and 25% for the low-volume (<15 cm^3^), intermediate-volume (15–70 cm^3^), high-volume (>70–130 cm^3^) and very high-volume group (>130 cm^3^), respectively (*p* = 0.007, see Fig. 2a-b). Corresponding 5-year RFS rates for the prognostic volume groups were 88%, 74%, 62%, and 25%, respectively.

There was also a significant correlation of tumor volume with OS(*p* < 0.001), with 3-year OS rates for the prognostic volume groups of 87%, 79%, 74% and 17%, respectively, and 5-year OS of 87%, 71%, 69% and 17%, respectively.

Using the upper quartile of 4.68 as cut-off value for further analysis, the subgroup with elevated NLR showed a significantly reduced RFS and OS with a difference for RFS at 3-years of 44% vs. 81% (*p* < 0.001) and at 3-years for OS of 56% vs. 84% (*p* < 0.001), respectively (Fig. 2c-d).

**Fig. 3:**
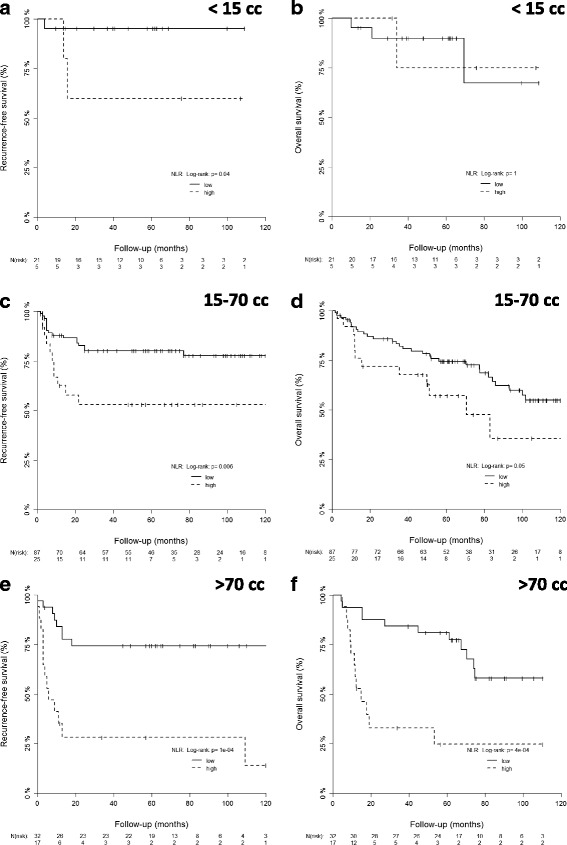
Stratification for NLR in different tumor volume groups. Patients with elevated NLR (> = 4.68) showed a significantly reduced recurrence-free survival in all tumor volume groups as well as inferior overall survival in the intermediate and high-tumor volume group (**a**-**b**: <15 cm^3^, **c**-**d**: 15-70 cm^3^, **e**-**f**: >70 cm^3^). NLR resulted in an additional statistically significant prognostic differentiation of the volumetric cohorts with respect to RFS and OS rates (except of the ‘small tumor volume’ cohort with only 4 events, Fig. 3b)

The NLR remained a significant prognostic factor when used for each volume group separately: Patients with elevated NLR (> = 4.68) showed a significantly reduced recurrence-free survival in all tumor volume groups (15 cm^3^, 15-70 cm^3^, >70 cm^3^) as well as a significantly inferior overall survival in the high-tumor volume group and a trend towards significance for the intermediate volume group (Fig. 3). Three-year OS and RFS for all tumor volume groups with and without elevated NLR is summarized in Table 2.

**Table 2:** Prognostic value of the NLR in different tumor volume risk groups. Cut-off for the NLR was 4.68

	3-year recurrence-free survival				3-year overall survival			
	High NLR	Low NLR	Hazard ratio	*P* value	High NLR	Low NLR	Hazard ratio	*P* value
Small tumor volume (< 15 cm^3^)	60%	95%	8.16	**0.041**	75%	90%	0.95	0.964
Intermediate tumor volume (15–70 cm^3^)	53%	80%	2.77	**0.006**	68%	82%	1.95	0.052
High tumor volume (>70 cm^3^)	28%	74%	5.04	**< 0.001**	33%	84%	4.16	**< 0.001**

### Univariate and multivariate regression analysis

Univariate analysis showed significantly increased hazard ratios for OS and RFS for elevated NLR and tumor volume, and a significantly reduced hazard ratio for normal ECOG, and the absence of smoking history. The application of full-dose cisplatin chemotherapy (≥ 200 mg per square meter body surface total dose) was significantly associated only with OS, and showed a trend towards significance for RFS (*p* = 0.052, Table 3).

**Table 3:** Univariate Analysis for recurrence-free survival (RFS) and overall survival (OS)

	RFS			OS		
	HR	Conf. int.	*p*-value	HR	Conf. int.	*p*-value
Age	1.001	0.9729–1.03	0.942	1.018	0.9932–1.043	0.157
Sex	0.7161	0.3755–1.366	0.308	0.755	0.4312–1.321	0.323
Normal ECOG	0.3812	0.213–0.6823	**0.002**	0.333	0.2009–0.5518	**< 0.001**
UICC stage	1.14	0.6254–2.079	0.668	1.163	0.6781–1.996	0.582
No smoking history	0.2293	0.05573–0.9431	**0.026**	0.314	0.1142–0.8635	**0.018**
Chemotherapy (full dose)	0.5653	0.3158–1.012	0.052	0.555	0.335–0.9195	**0.021**
Tumor volume	1.015	1.008–1.021	**< 0.001**	1.011	1.005–1.017	**< 0.001**
NLR	4.059	2.315–7.117	**< 0.001**	2.311	1.438–3.714	**< 0.001**

For multivariate Cox regression analysis, all significant factors from univariate analysis were included. Tumor volume, elevated NLR and ECOG status remained significant on multivariate testing, whereas chemotherapy and smoking status did not (Table 4).

**Table 4:** Multivariate analysis for recurrence-free survival (RFS) and overall survival (OS)

	RFS			OS		
	HR	Conf. int.	*p*-value	HR	Conf. int.	*p*-value
Normal ECOG	0.5212	0.28515 0.9526	0.521	0.4027	0.2384 0.6804	**< 0.001**
No smoking history	0.4191	0.09899 1.7739	0.237	0.4887	0.1733 1.3786	0.176
Chemotherapy (full dose)	0.9337	0.50318 1.7328	0.828	0.8130	0.4757 1.3892	0.449
Tumor volume	1.0111	1.00454 1.0177	**0.036**	1.0077	1.0016 1.0138	**0.013**
NLR	3.0218	1.67305 5.4579	**< 0.001**	1.7333	1.0462 2.8718	**0.033**

## Discussion

Volumetric tumor staging has been previously established by our group and others as superior prognostic factor compared to the TNM and UICC staging systems for patients with locally advanced head and neck cancer undergoing IMRT [3–5]. As previously reported, we have identified distinct cut-off values for volumetric staging in a prospective patient cohort which correlate well with recurrence-free survival and overall survival [3]. However, there is still a considerable difference in oncological outcome within the pre-defined volume groups, which supports the use of additional prognostic factors such as HPV status [27], advanced imaging [28] or clinical immunoscores [15, 29] to determine the individual risk group of a patient.

Our aim was to analyze the prognostic impact of the NLR in addition to the previously established volumetric risk groups in a cohort of patients undergoing definitive radio(chemo)therapy for oropharyngeal cancer. Our hypothesis was that the use of the NLR may further refine the prognostic volumetric groups which could be confirmed based on a significant association with RFS in all volume groups and with OS in the high tumor volume group. While several authors have investigated the role of the NLR alone in head neck cancer [12, 13, 15], this study is, to our knowledge, the first analysis which combines the prognostic factors of tumor volume and the NLR.

Our data of a large non-surgical cohort of OC treated with IMRT confirms the findings of other groups [12, 13, 15] that one of the most commonly investigated immunoscores, the NLR, was significantly associated with recurrence-free survival and overall survival (see Table 5). Although the NLR showed a weak, but statistically significant correlation with tumor volume, the NLR remained a significant independent prognostic marker in all tumor volume subgroups.

**Table 5:** Summary of studies investigating the prognostic role of the neutrophil-lymphocyte ratio (NLR) in oropharyngeal cancer (OC)

Study	Cohort and treatment	NLR cut-off	Results	p16 status
Rachidi et al. [13]	*n* = 543 HNSCC (170 OC), any treatment (2000–2012)	4.39 (upper tertile)	- Increased mortality for high NLR (HR = 2.39) - NLR prognostic factor both in p16-pos. And neg. Pts. - NLR significantly lower in p16-pos. Patients	yes (89/543)
Charles et al. [29]	*n* = 145 (76 OC), radio(chemo)therapy (2005–2012)	5.0 (based on review [39])	- High NLR associated in OC with inferior OS (HR = 4.6) and RFS (HR = 3.01) - No subgroup analysis for p16 pos. Pts.	yes (95/145)
Kano et al. [40]	*n* = 285 HNSCC (116 OC), radiochemotherapy (2003–2012)	1.92 (based on ROC analysis)	- High NLR associated with inferior OS and DFS, but not significant on multivariate analysis	no
Valero et al. [35]	*n* = 824 (203 OC), any treatment (2010–2012)	1.35 and 3.86 (three groups based on RPA)	- High NLR associated with inferior DSS - Lower neutrophil number in p16 pos. Pts.	yes (125/824)
Selzer et al. [14]	*n* = 170 (74 OC), primary radio(chemo)therapy or radioimmunotherapy (2002–2012)	5.0	- High NLR associated with inferior median OS (17 vs. 27 months)	no
Moon et al. [41]	*n* = 153 (51 OC), HNSCC prospective study (2010–2012)	not described	- High NLR associated with inferior PFS (HR = 2.20) and OS (HR = 3.22)	no
Huang et al. [34]	*n* = 510, OC, radio(chemo)therapy (2000–2010)	not applied (neutrophils and lymphocytes were analyzed separately)	- High neutrophils and low lymphocytes are associated with inferior prognosis - Reduced neutrophil count and similar lymphocyte count in p16-pos. Pts.	yes (all)
Young et al. [42]	*n* = 249, OC, radio(chemo)therapy (2004–2010)	5.0	- High NLR associated with inferior locoregional control (HR = 2.072)	no

*HNSCC* squamous cell carcinoma of the head and neck, *pts.* patients, *ROC* receiver-operating characteristic, *RPA* recursive partitioning analysis, *OS* Overall survival, *PFS* progression-free survival, *DFS* disease-free survival, *DSS* disease-specific survival

Sun et al. [15] previously demonstrated the prognostic significance of NLR in different UICC-stage-based subgroups in nasopharyngeal cancer with no significant impact in stage I and II disease. Similarly, our data for OC shows a significant correlation with RFS, but not with OS in small tumor volumes (<15 cm^3^), which, however, may also be due to the small sample size (*n* = 26) and the limited number of events (*n* = 4).

Our study was limited by the following facts:

- While the tumor volume was assessed prospectively since 2004, the NLR was collected retrospectively from electronic patient records and blood samples were not taken systematically for this purpose at a specific time point.
- A general limitation of the NLR is that is affected by any inflammatory condition such as infections or invasive procedures as well as by myelosuppressive conditions such as (induction) chemotherapy which led to the exclusion of several patients in our analysis. Future clinical immunoscores may therefore include more cancer-specific hematological markers and identify specific leucocyte subgroups. For instance, it was found that head and neck cancer patients showed an increased number of immature granulocytes in the peripheral blood [30] as well as unique immunophenotypes of immunosuppressive neutrophils (CD11c bright/CD62L dim/CD11b bright/CD16 bright), which were found in cancer patients but not in healthy donors [31].
- The major limitation of our study is the fact that included patients were not systematically screened for HPV infection, which has been recently established as strong prognostic factor in OC [27, 32, 33], but which had not yet been established as standard at our institution in the investigated period (2002–2011). A subset analysis of OC patients with available p16 status has recently shown that p16-positive patients presented with significantly lower NLR, but the NLR remained a significant prognostic factor both in the p16-positive and p16-negative group [13]. Additionally, other studies confirmed blood neutrophils and lymphocytes as strong prognostic factors in p16-positive OC with significantly lowered blood neutrophils compared to the p16-negative group [34, 35]. These findings are complemented by current immunological research in head and neck cancer which suggests an increased anti-tumor immunity, particularly increased numbers of tumor-infiltrating lymphocytes in p16 positive tumors [36]. Future studies on larger OC cohorts including p16 status will have to clarify the role and correlation of p16 status and NLR as prognostic factors.

Recent advances in cancer research have identified inflammatory response as crucial factor for tumor development and progression, and, on the other hand, the presence of tumor-infiltrating lymphocytes as positive prognostic markers in several tumor entities [37]. The NLR is a clinical immunoscore which can be easily deducted from a differential blood count, which is frequently available in patients receiving chemoradiotherapy. It has been established as prognostic marker in several tumor entities [24] including several cohorts of different head and neck cancer subgroups treated with surgery or radiotherapy [12, 15, 17, 38].

We decided to use a single, representative cut-off value for the NLR instead of a subdivision into several prognostic groups, as our major aim was to demonstrate *in principle* the prognostic impact of the NLR in each prognostic tumor volume group. The cut-off value for the NLR in this study of 4.68 is in the same range as in previous publications on NLR in OC (see Table 5). Additionally, a further subdivision with several NLR cut-offs would have resulted in too small subgroups in our single institution cohort which may not allow to draw a significant conclusion.

## Conclusion

In summary, our results demonstrate that the NLR is an independent prognostic factor for patients with OC undergoing radio(chemo)therapy regarding RFS in all tumor volume subgroups and regarding OS in high-volume groups. The NLR and tumor volume represent two easily available clinical parameters that impose no additional diagnostic burden to the patients. Future prospective studies are needed to validate our findings. In addition, blood assays are needed that identify more specific subtypes of circulating leukocytes in order to improve the accuracy of oncological immunoscores.

## Abbreviations

CT: Computed tomography
DFS: Disease-free survival
DSS: Disease-specific survival
ECOG: Eastern Cooperative Oncology Group
HNSCC: Squamous cell carcinoma of the head and neck
HPV: Human papilloma virus
IMRT: Intensity-modulated radiotherapy
NLR: Neutrophil-lymphocyte ratio
OC: Oropharyngeal cancer
OS: Overall survival
PET: Positron emission tomography
RFS: Recurrence-free survival
ROC: Receiver operating characteristic
tGTV: Total gross tumor volume
UICC: Union for International Cancer Control
